# The impact of culture systems on the gut microbiota and gut metabolome of bighead carp (*Hypophthalmichthys nobilis*)

**DOI:** 10.1186/s42523-023-00239-7

**Published:** 2023-04-01

**Authors:** Chen Ye, Shiyu Geng, Yingyu Zhang, Huimin Qiu, Jie Zhou, Qi Zeng, Yafei Zhao, Di Wu, Guilan Yu, Haibo Gong, Beijuan Hu, Yijiang Hong

**Affiliations:** 1 School of Life Science, Nanchang University, Nanchang, 330031 China; 2 Jiangxi Province Key Laboratory of Aquatic Animal Resources and Utilization, Nanchang University, Nanchang, 330031 China; 3 Jiangxi Provincial Aquatic Biology Protection and Rescue Center, Nanchang, 330000 China; 4 Modern Agricultural Research Institute, Nanchang University, Nanchang, 330031 China

**Keywords:** Culture system, Gut microbiota, Metabolome, Bighead carp, Fish muscle quality

## Abstract

**Background:**

The gut microbiota of fish confers various effects on the host, including health, nutrition, metabolism, feeding behaviour, and immune response. Environment significantly impacts the community structure of fish gut microbiota. However, there is a lack of comprehensive research on the gut microbiota of bighead carp in culture systems. To demonstrate the impact of culture systems on the gut microbiome and metabolome in bighead carp and investigate a potential relationship between fish muscle quality and gut microbiota, we conducted a study using 16S ribosomal ribonucleic acid sequencing, gas chromatography-mass spectrometry, and liquid chromatography-mass spectrometry techniques on bighead carp in three culture systems.

**Results:**

Our study revealed significant differences in gut microbial communities and metabolic profiles among the three culture systems. We also observed conspicuous changes in muscle structure. The reservoir had higher gut microbiota diversity indices than the pond and lake. We detected significant differences in phyla and genera, such as Fusobacteria, Firmicutes, and Cyanobacteria at the phylum level, *Clostridium sensu stricto 1*, *Macellibacteroides*, *Blvii28 wastewater sludge group* at the genus level. Multivariate statistical models, including principal component analysis and orthogonal projections to latent structures-discriminant analysis, indicated significant differences in the metabolic profiles. Key metabolites were significantly enriched in metabolic pathways involved in "arginine biosynthesis" and "glycine, serine, and threonine metabolism". Variation partitioning analysis revealed that environmental factors, such as pH, ammonium nitrogen, and dissolved oxygen, were the primary drivers of differences in microbial communities.

**Conclusions:**

Our findings demonstrate that the culture system significantly impacted the gut microbiota of bighead carp, resulting in differences in community structure, abundance, and potential metabolic functions, and altered the host's gut metabolism, especially in pathways related to amino acid metabolism. These differences were influenced substantially by environmental factors. Based on our study, we discussed the potential mechanisms by which gut microbes affect muscle quality. Overall, our study contributes to our understanding of the gut microbiota of bighead carp under different culture systems.

**Supplementary Information:**

The online version contains supplementary material available at 10.1186/s42523-023-00239-7.

## Background

The gut microbiota in fish has been proven to have significant effects on the health [1, 2], nutrition [3, 4], and metabolism of the host [5, 6].

The fish gut microbiota is shaped by environmental factors, including microbial community structure [7], diversity, and richness [8]. Previous studies have reported significant alterations in the intestinal microbial communities of various fish species due to environmental factors [9–11].

Our previous study [12] showed significant differences in bighead carp muscle fatty acids, amino acids, and volatile flavour compounds in different aquaculture systems, suggesting that the culture systems could affect muscle quality. However, the potential relationship between the culture systems and muscle quality needs further exploration [12], where gut microbes may play a crucial role. Studies have reported the impact of gut microbiota on muscle quality in other species [13, 14]. However, little research has been done to explore the microbiota's role in improving fish muscle quality.

Bighead carp (*Hypophthalmichthys nobilis*), which belongs to the order Cypriniformes, family Cyprinidae, subfamily Hypophthalmichthyinae, and genus *Hypophthalmichthys* [15], is referred to China as one of the "four major domesticated fish" [16]. It is an omnivorous filter-feeding fish [17] that is highly popular in China due to its affordability, delicious taste, and high nutritional value [18]. It is also one of the world's most important aquaculture species [19], with an output of 3.187 million tons, accounting for 6.5% of the world's inland finfish production [20]. Previous studies have mainly focused on the gut microbiota of bighead carp in ponds [21–26], with limited research conducted in lakes [16, 27–30] and reservoirs [28–30]. There is a lack of comprehensive studies on the gut microbiota of bighead carp in conventional culture systems.

Therefore, this study aimed to investigate the impact of three typical culture systems on the gut microbiota, gut metabolome, and muscle structure of bighead carp. In addition, we explored the potential mechanisms by which gut microbes and gut metabolites drive changes in muscle quality in bighead carp.

## Methods

### Fish, culture systems, and sampling

Two-year-old bighead carp with a body length of 35.0 ± 3.70 cm, body width of 7.8 ± 0.6 cm, and body weight of 1.20 ± 0.10 kg were purchased from the same breeding farm and cultured in the three culture systems in March 2016: NC (Nancheng), which is a culture pond; PY (Poyang), which represents the inner lake of Poyang Lake, the largest freshwater lake in China; and XHK (Xiahuikeng), which is an alpine cold-water reservoir at 420 m. The water surface areas of NC, PY, and XHK are 0.53, 40, and 90 ha, respectively.

Bighead carp in NC were fed artificially, while fish in PY and XHK were free-range. The biochemical composition of the formulated feed in NC was moisture ≤ 12.0%, crude protein ≥ 28.0%, crude fibre ≤ 11.6%, crude ash ≤ 15.0%, crude lipid ≥ 3.5%, total phosphorus ≥ 0.7%, and lysine ≥ 1.3%.

A total of 30 bighead carp representing three groups with ten replicates were randomly caught and anaesthetized with an overdose of tricaine methane sulfonate in March 2017. The hindgut content of bighead carp was extracted under aseptic conditions, washed with phosphate buffer saline, placed in sterile centrifuge tubes, frozen in liquid nitrogen until fully frozen, and then stored at − 80 °C for deoxyribonucleic acid (DNA) and metabolite extraction. The white epaxial muscle was cut transversely into 0.5 × 0.5 cm blocks and fixed in formalin for 24 h.

### Environmental factors measurement

During the cultural period, environmental factors of the culture systems were tracked every season. Water temperature (WT), dissolved oxygen (DO), pH, nitrate nitrogen (NO_3_-N), and ammonium nitrogen (NH_4_-N) were measured by YSI Pro Plus multiparameter instrument. Transparency was obtained by measuring the maximum visible depth of the Secchi disk underwater.

### Muscle histology

Fixed blocks were embedded in paraffin, sectioned, and stained with hematoxylin and eosin [31]. Tissue images were photographed using a Nikon DS-Ri2 microscope camera. Muscle cells from the image of each sample were segmented with Cellpose 2.0 software [32]. The long and short diameters of muscle fibres were measured with Fiji [33].

### DNA extraction, 16S ribosomal ribonucleic acid rRNA (16S rRNA) gene sequencing, and data processing

According to the instruction manual, DNA from intestinal contents was extracted using a DNeasy PowerSoil Kit (QIAGEN, Germany). The concentration and integrity of DNA were measured with a NanoDrop 2000 spectrophotometer (NanoDrop, USA) and agarose gel. The V3–V4 hypervariable region of the bacterial 16S rRNA gene was amplified by polymerase chain reaction (PCR) with universal primer pairs (343F/798R) [34]. The amplicon quality was visualised using gel electrophoresis. The PCR products were purified with Agencourt AMPure XP beads (Beckman Coulter, USA) and quantified using a Qubit dsDNA assay kit. Sequencing was performed on an Illumina NovaSeq 6000 (Illumina, USA).

The raw image data file was transformed into raw sequence data through base recognition analysis. Paired-end reads were preprocessed using Trimmomatic software [35], which removed ambiguous bases and low-quality sequences with average quality scores below 20 using a sliding window trimming approach. Paired-end reads were then assembled using fast length adjustment of short reads software [36]. Reads with 75% of bases above Q20 were retained, chimaeras were removed, and valid tags were obtained using quantitative insights into microbial ecology (QIIME) software [37]. Clean reads were subjected to primer sequence removal and clustered to generate operational taxonomic units (OTUs) with a 97% similarity cutoff using Vsearch software [38].

All representative reads selected by QIIME were annotated and blasted against the Silva database (v123) [39] using the ribosomal database project naïve Bayesian classifier (confidence threshold: 70%) [40]. The resulting OTU abundance matrix and annotation information were exported for downstream analysis.

In MicrobiomeAnalyst [41], the OTU data were filtered and rarefied to the minimum library size. Bacterial alpha diversity was assessed using the Shannon, Simpson, Chao1, and ACE indices. The distance matrix was calculated using non-metric multidimensional scaling (NMDS) [42] based on Bray-Curtis distance and visualised by the first two coordinates in NMDS. A one-way analysis of similarity (ANOSIM) [43, 44] with 999 permutations was performed to test for the significant difference between groups using the R vegan [45] and ecodist package [46]. Linear discriminant analysis effect size (LEfSe) [47] was applied to identify differences in taxa composition. The taxa with the logarithm to the base 10 of linear discriminant analysis (LDA) score > 2.0 and false discovery rate (FDR) (Kruskal-Wallis test) < 0.05 were regarded as differential taxa. The phylogenetic investigation of communities by reconstruction of unobserved states (PICRUSt2) [48] was applied to predict bacterial function.

### Metabolite extraction

40 μL of internal standards (2-chloro-l-phenylalanine in methanol, 0.3 mg/mL, 20 μL; lysophosphatidylcholine 17:0 in methanol, 0.01 mg/mL, 20 μL), 600 μL of extraction solvent with methanol/water (4/1, volume/volume) and steel balls were added to the 60 mg sample. The samples were stored at − 20 °C, ground at 60 Hz for 2 min, ultrasonicated for 10 min, held at − 20 °C for 30 min, centrifuged at 13,000 rpm, and 4 °C for 10 min. Then, 300 μL of supernatant was collected and dried in a freeze-concentration centrifugal dryer, redissolved in a 400 μL mixture of methanol and water (1/4, volume/volume), vortexed, ultrasonicated, and centrifuged again. Finally, 150 μL of supernatant was collected via syringes, filtered through 0.22 μm microfilters, and transferred to vials for liquid chromatography-mass spectrometry (LC-MS) analysis.

The 60 mg sample was supplemented with 40 μL of internal standard (2-chloro-l-phenylalanine in methanol, 0.3 mg/mL) and 360 μL of cold methanol. After grinding and ultrasonication at 4 °C for 30 min, 200 μL of chloroform was added to each sample and vortexed at 60 Hz for 2 min. This was followed by the addition of 400 μL of water, another round of vortexing, ultrasonication at 4 °C for 30 min, storage at − 20 °C for 30 min, and centrifugation for 10 min. Next, 300 μL of supernatant was transferred to a vial and dried. To the sample, 80 μL of methoxylamine hydrochloride (in pyridine, 15 mg/mL) was added, vortexed for 2 min, and incubated at 37 °C for 90 min. A mixture of 80 μL N, O-Bis(trimethylsilyl)trifluoroacetamide (with 1% trimethylchlorosilane), 20 μL n-hexane and 10 μL fatty acid methyl esters (C8/C9/C10/C12/C14/C16, 0.8 mg/mL; C18/C20/C22/C24/C26, 0.4 mg/mL; all in chloroform) was then added to each sample, vortexed for 2 min and then derivatised at 70 °C for 60 min for gas chromatography-mass spectrometry (GC-MS) analysis.

A quality control (QC) sample was prepared by mixing aliquots of all samples. The QC sample was injected every ten runs for assessment of data repeatability.

### LC–MS analysis

The derivatised samples were analysed using a Dionex Ultimate 3000 RS HPLC system equipped with a Q-Exactive quadrupole-Orbitrap mass spectrometer, which had a heated electrospray ionisation source (Thermo Fisher, USA). The ACQUITY UPLC HSS T3 column (1.8 μm, 2.1 × 100 mm, Waters, USA) was used, and the binary gradient elution system consisted of (A) water (containing 0.1% formic acid, volume/volume) and (B) acetonitrile (containing 0.1% formic acid, volume/volume). Separation was achieved using the following parameters: 0–1 min, 5% B; 1–11 min, 5–100% B; 11–13 min, 100% B; 13–13.1 min, 100% to 5% B; 13.1–15 min, 5% B; flow rate, 0.35 mL/min; column temperature, 50 °C; injection volume, 5 μL.

The mass spectrometer was operated as the following parameters: mass range, *m*/*z* 70–1000; resolution for the full mass spectrum (MS) scan and MS/MS scans, 70,000 and 17,500, respectively; normalised collision energy and stepped normalised collision energy, 20 eV and 40 eV, respectively; spray voltage, 3800 V (positive) and 3000 V (negative); sheath gas flow rate, 35 arbitrary units; auxiliary gas flow rate, 8 arbitrary units; capillary temperature, 320 °C; Aux gas heater temperature, 350 °C; S-lens RF level, 50 V.

Raw data were collected using UNIFI 1.8.1 software and analysed using Progenesis QI 2.3 software (Waters, USA) with baseline filtering, retention time correction, peak recognition, peak alignment, and integration operations. Isotopic peaks were excluded, and the minimum intensity was set to 15% of the base peak intensity. Metabolites were identified based using the Human Metabolome Database [49], LIPIDMAPS (v2.3) [50], and Metabolite Link [51], based on the exact mass number, secondary mass fragment, and isotope distribution.

Then, the data matrix was outputted with three-dimensional datasets, including *m*/*z*, retention time, and peak intensities. The matrix was reduced by removing metabolites with more than 50% missing values, uncertain metabolites, and metabolites with a relative standard deviation (RSD) > 0.4 in QC samples. Then, the remaining missing values were imputed by half of the minimum value. All metabolites were segmented and normalised based on internal standards, and then the internal standards were removed.

### GC–MS analysis

The derivatised samples were separated and analysed on an Agilent 7890B gas chromatography system coupled to an Agilent 5977A MSD system with a DB-5MS column (30 m × 0.25 mm × 0.25 μm) (Agilent, USA). The conditions were set as follows: carrier gas, helium; flow rate, 1 mL/min; injector temperature, 260 °C; injection volume, 1 μL by splitless mode. quadrupole temperature, 150 °C; electron impact ion source, 230 °C. The initial oven temperature was 60 °C, ramped to 125 °C at a rate of 8 °C/min, to 210 °C at a rate of 5 °C/min, to 270 °C at a rate of 10 °C/min, to 305 °C at a rate of 20 °C/min, and finally held at 305 °C for 5 min. The ionisation energy was 70 eV. Mass data were acquired in full-scan mode (*m*/*z* 50–500), and the solvent delay time was 5 min.

The raw data were converted using Analysis Base File Converter software and processed via Mass Spectrometry-Data Independent Analysis software for peak detection, deconvolution, alignment, and filtering. Metabolites were annotated using a database from Lumingbio company (China). Internal standard ion peaks with RSD > 0.3 were removed, and all peaks were segmented and normalised based on the internal standard and fatty acid methyl esters. All fatty acid methyl esters, internal standards, and known pseudo-positive peaks were then removed. The resulting data matrix was exported, including sample information, peak names, and peak intensities.

### Metabolomics analysis

LC-MS and GC-MS data matrixes were combined and imported into Metaboanalyst [52]. The data were transformed and scaled, and then principal component analysis (PCA) and orthogonal projections to latent structures-discriminant analysis (OPLS-DA) [53, 54] were performed. The quality of the OPLS-DA models was evaluated with R^2^X and Q^2^. Permutation tests with 1000 permutations were carried out to assess the fitting of the models. Variable importance in projection (VIP) of metabolites was calculated in the OPLS-DA models.

Metabolites with VIP value ≥ 1.0 and *p* value < 0.05 were considered significantly differential, and those present in all intergroup comparisons were considered key. Metabolic pathway analysis (MetPA) was carried out on these metabolites, and enriched pathways with *p *value < 0.05 were considered significant.

### Association analysis

To investigate potential associations between significant gut microbes and metabolites, we used the R psych package [55]. Spearman rank correlation was applied to filter associated microbes and metabolites with *p* value < 0.05 and *r* > 0.8.

Variation partitioning analysis (VPA) was performed using the R vegan package to examine the relative importance of environmental factors for gut microbiota variation. Redundancy analysis (RDA) with 999 permutations was carried out to correlate significantly differential microbes at the phylum and genus levels with environmental factors based on the first axis length of detrended correspondence analysis calculated by the R vegan package.

### Statistical analysis

Data analysis was performed using R 4.2.0 [56] and SPSS 26.0 (SPSS Inc.). The Wilcoxon test and Kruskal-Wallis test were used for significance testing. Multiple comparisons and the Benjamini-Hochberg method correction were performed when the significance in the Kruskal-Wallis test was less than 0.05. *p* value or FDR < 0.05 was considered statistically significant. We denoted significance levels with *, **, and *** representing *p* value or FDR < 0.05, 0.01, and 0.001 between groups, respectively. Values were expressed as mean ± standard deviation.

## Results

### Muscle structure

**Fig. 1:**
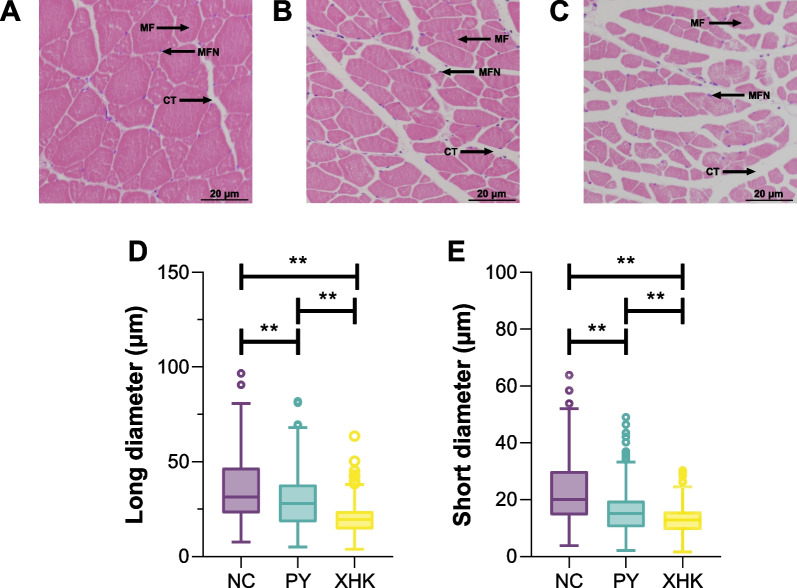
Transections of muscle tissues and muscle fibres parameters of bighead carp from three groups. **A** NC × 200; **B** PY × 200; **C** XHK × 200; **E** long diameter of muscle fibres; **F** short diameter of muscle fibres. (MF: muscle fibre; MFN: muscle fibre nucleus; CT: connective tissue). Significance levels with *, **, and *** represent FDR < 0.05, 0.01, and 0.001 between groups, respectively (Kruskal-Wallis test)

Significant differences were observed in the muscle structure of the three groups of bighead carp. The NC group (Fig. 1A) showed tightly arranged muscle fibres, less connective tissue, and larger myocytes. The PY group (Fig. 1B) and XHK group (Fig. 1C) had loosely aligned muscle fibre, abundant connective tissue, and smaller myocytes.

The long and short diameters of the muscle fibres were also significantly different among all three groups, with NC having the largest, PY the second largest, and XHK the smallest (Kruskal-Wallis, FDR < 0.05) (Fig. 1D, E).

### Microbial community structure of the gut in bighead carp

The processed data are shown in Additional file 5: Table S1. The microbiota was classified into 19 phyla, 33 classes, 75 orders, 128 families, 172 genera, and 13 species.

**Fig. 2:**
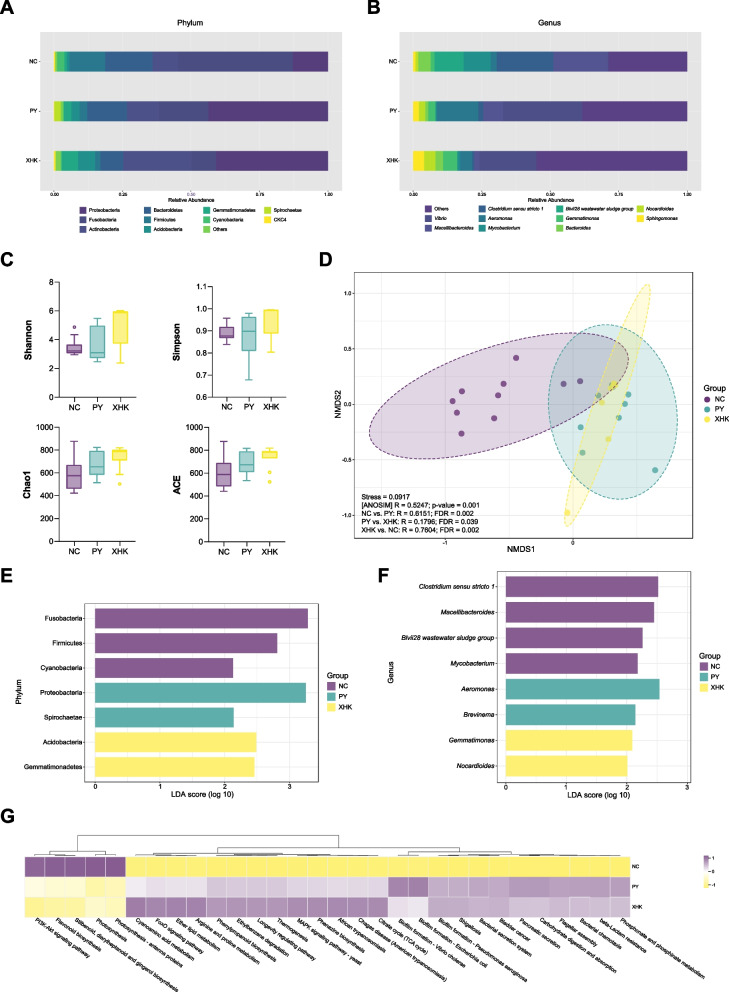
The composition, alpha diversity, beta diversity, and indicator of gut microbiota. **A** The composition and relative abundance of the top 10 phyla. The bars with different colours represent different phyla. **B** The composition and relative abundance of the top 10 genera. The bars with different colours represent different genera. **C** The three groups' diversity indices (Shannon, Simpson, Chao1, and ACE). The outliers were calculated by Tukey's test. The horizontal lines of the boxplot, from top to bottom, represent the maximum value except for outliers, upper quartile, median, lower quartile, and minimum value except for outliers, respectively. **D** Every point in the plot represents a sample. NMDS plot shows a clear separation of every two groups. ANOSIM demonstrates that culture systems significantly impacted the gut bacterial community composition (R > 0 indicates that the difference between groups is greater than within groups, and FDR < 0.05 indicates that the difference is significant). **E**, **F** LEfSe analysis: The log LDA score of gut microbiota at the phylum and genus levels (log LDA score > 2.0). The bars with different colours represent the significant phyla or genera of the corresponding group. **G** The heatmap of the top 20 significant pathways predicted by PICRUSt2 (FDR < 0.05). The boxes with purpler colours represent higher expression levels, and yellower colours represent lower expression levels

At the phylum level (Fig. 2A), the different culture systems had similar community structures but differed in abundance. Proteobacteria and Acidobacteria were significantly higher in PY and XHK than in NC (Kruskal-Wallis test, FDR < 0.05), while Fusobacteria, Firmicutes, and Cyanobacteria showed the opposite trend (Kruskal-Wallis test, FDR < 0.05). The abundance of Gemmatimonadetes was significantly higher in XHK than in NC (Kruskal-Wallis test, FDR < 0.05), and Spirochaetae was significantly higher in PY than in NC (Kruskal-Wallis test, FDR < 0.05) (Additional file 6: Table S2).

At the genus level (Fig. 2B), there were significant differences in the gut microbial communities. For example, the abundance of *Macellibacteroides* was significantly higher in NC than in XHK (Kruskal-Wallis test, FDR < 0.05). *Clostridium sensu stricto 1*, *Mycobacterium,* and *Blvii28 wastewater sludge groups* were significantly more abundant in NC (Kruskal-Wallis test, FDR < 0.05) than in the other groups, while *Nocardioides* and *Sphingomonas* were significantly less abundant (Kruskal-Wallis test, FDR < 0.05) (Additional file 7: Table S3).

The diversity indices of the gut bacteria, such as Shannon, Simpson, Chao1, and ACE, are shown in Fig. 2C. Overall, the diversity indices in XHK were higher than those in NC and PY. However, there were no significant differences among groups in Shannon (Kruskal-Wallis test, *p* = 0.063), Simpson (Kruskal-Wallis test, *p* = 0.070), Chao1 (Kruskal-Wallis test, *p* = 0.093) and ACE (Kruskal-Wallis test, *p* = 0.155), which indicated that the impact of culture systems on bacterial diversity was limited.

We performed NMDS analysis based on the Bray–Curtis distance to analyze differences in bacterial communities among groups, as shown in Fig. 2D. NC was well separated from the other groups, and PY had a microbial community similar to that of XHK. The stress value (0.0917 < 0.1) indicated a stable NMDS model. ANOSIM and multiple comparisons results indicated that every two groups separated significantly (NC vs. PY, FDR = 0.002; PY vs. XHK, FDR = 0.039; XHK vs. NC, FDR = 0.002), and the culture systems had a significant impact on the bacterial community (ANOSIM: R = 0.5247; *p* = 0.001).

To determine the indicator taxa among groups, we applied LEfSe and presented the results at the phylum and genus levels in Fig. 2E, F, respectively. The results indicated 7 phyla and 8 genera with a significant difference among the groups (log LDA score > 2.0, Kruskal-Wallis test, FDR < 0.05). At the phylum level, Fusobacteria, Firmicutes, and Cyanobacteria were significant in NC; Proteobacteria and Spirochaetae were significant in PY; and Acidobacteria and Gemmatimonadetes were significant in XHK (Additional file 1: Fig S1). At the genus level, the indicators were *Clostridium sensu stricto 1*, *Macellibacteroides*, *Blvii28 wastewater sludge group* and *Mycobacterium* in NC; *Aeromonas* and *Brevinema* in PY; and *Gemmatimonas* and *Nocardioides* in XHK (Additional file 2: Fig S2)*.*

PICRUSt2 was utilized to predict bacterial function in the three groups. A total of 370 pathways were predicted, with 278 pathways identified as significant (Kruskal-Wallis test, FDR < 0.05), indicating that culture systems affected the potential metabolic capacity of gut microbiota. Among the significant pathways, almost half of the pathways were categorized under "metabolism" (131/278, 47.12%). Only a few pathways were classified under "organismal systems" (49/278, 17.63%), "human diseases" (43/278, 15.47%), "cellular processes" (24/278, 8.63%), "environmental information processing" (22/278, 7.91%), and "genetic information processing" (9/278, 3.24%). The top 20 pathways, ranked by *p*-value, are shown in Fig. 2G, with the most altered pathways being "cyanoamino acid metabolism", "photosynthesis", and "phenylpropanoid biosynthesis".

### Metabolic profiling of the gut in bighead carp

A total of 1453 metabolites were detected, with 1168 identified by LC-MS and 285 by GC-MS. These metabolites included 681 lipids and lipid-like molecules, 184 organic acids and derivatives, 95 organic oxygen compounds, 76 organ heterocyclic compounds, 61 benzenoids, 33 phenylpropanoids and polyketides, etc. (Additional file 8: Table S4).

**Fig. 3:**
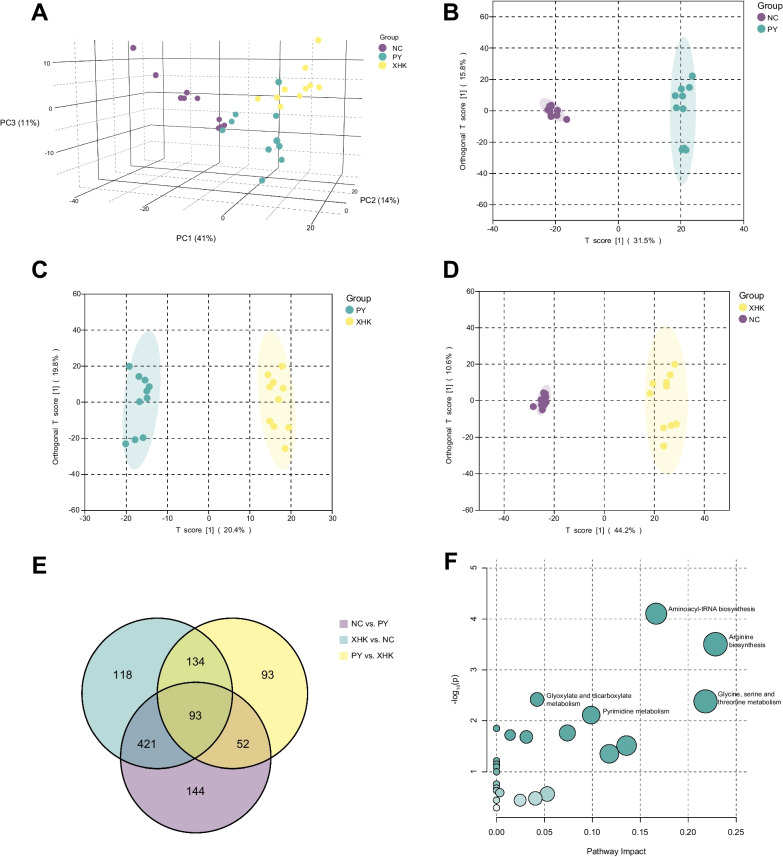
Multivariate statistical analysis and metabolic pathway analysis of intestinal contents. **A** The PCA score plot clearly separates NC, PY, and XHK, indicating different metabolic profiles among the three groups. **B**–**D** The OPLS-DA score plots show a clear separation of every two groups, indicating strong effects of the culture systems on the metabolic composition. **E** The Venn diagram shows the number of significantly differential metabolites between groups, with the numbers in circles indicating the number of metabolites and the numbers in overlapping parts representing the number of common metabolites in intergroup comparison. **F** The enriched pathways are shown on a scatter plot, with the horizontal axis indicating the impact of the pathways and the vertical axis indicating the significance of the pathways. The significance and impact of each pathway are represented by the size and colour of the scatter, respectively

The data were subjected to PCA to compare the three groups' metabolic composition. The PCA score plot (Fig. 3A) could distinguish the three groups. Three principal components (PC1, PC2, and PC3) were extracted, explaining 41%, 14%, and 11% of the variability, respectively.

OPLS-DA was used to analyse the degree of variability in intergroup samples. High predictability (Q^2^) and strong goodness of fit (R^2^X, R^2^Y) were reflected between every two groups (Additional file 9: Table S5), which demonstrated that the models were stable and could be used to identify significantly differential metabolites further. In the OPLS-DA score plots (Fig. 3B–D), every two groups were clearly separated, indicating distinct differences in metabolic profiling between groups. Permutation tests indicated that the models were not overfitted (Additional file 9: Table S5).

Significantly differential metabolites were screened, revealing 710 metabolites in NC compared with PY, 372 metabolites in PY compared with XHK, and 766 metabolites in XHK compared with NC. A total of 93 common significantly differential metabolites were screened in all intergroup comparisons, which included 32 lipids and lipid-like molecules, 19 organic acids and derivatives, and 7 organic oxygen compounds (Fig. 3E).

MetPA was conducted on all these metabolites, which enriched 27 pathways, of which 11 were significant (hypergeometric test, *p* < 0.05) (Additional file 10: Table S6). These pathways included 4 related to amino acid metabolism, 3 to carbohydrate metabolism, 1 pathway to translation, 1 to nucleotide metabolism, 1 to lipid metabolism, and 1 to metabolism of cofactors and vitamins. The significantly altered pathways included “aminoacyl-tRNA biosynthesis", "arginine biosynthesis", "glyoxylate and dicarboxylate metabolism", "glycine, serine, and threonine metabolism", "pyrimidine metabolism" (Fig. 3F).

### The relationship between gut microbiota and metabolites

**Fig. 4:**
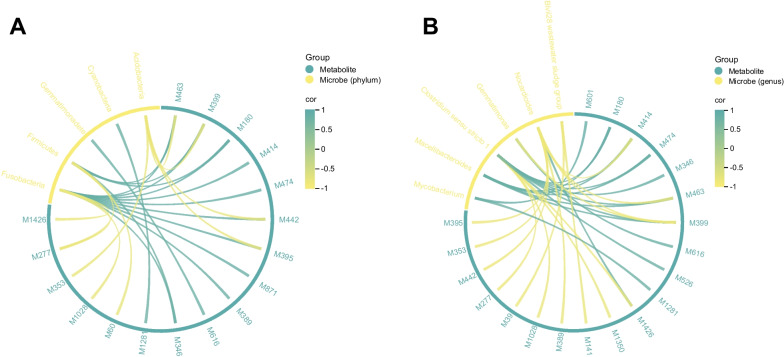
The correlation of microbes and metabolites. **A** The correlation at the phylum level. **B** The correlation at the genus level. The lines in the circles represent the correlation between metabolites and microbes, with greener colours representing more positive correlations and yellower colours representing more negative correlations

To explore potential associations between significant gut microbes and metabolites under environmental influence, we utilised Spearman rank correlation. At the phylum level, 26 associations between 17 metabolites and 5 microbes were found (Spearman rank correlation, *r* > 0.8, *p* < 0.05) (Fig. 4A). At the genus level, 30 associations were obtained for 6 microbes and 20 metabolites (Spearman rank correlation, *r* > 0.8, *p* < 0.05) (Fig. 4B). The details of the correlation can be seen in Additional file 11: Tables S7 and Additional file 12: Table S8.

### The correlation between environmental factors and gut microbiota

Table 1 shows the environmental factors, including water temperature (WT), dissolved oxygen (DO), nitrate nitrogen (NO_3_-N), ammonium nitrogen (NH_4_-N), pH, and transparency, for the three groups in four seasons. PY had the highest water temperature on average, significantly higher than the other two groups. NC had the highest levels of DO, NO_3_-N, NH_4_-N, pH, and transparency, with NH_4_-N content significantly higher than that in XHK, while other factors were significantly higher than the other groups (Kruskal-Wallis test, FDR < 0.05).

**Table 1:** Environmental factor parameters for the three groups in four seasons

	Spring (March)			Summer (August)			Autumn (October)			Winter (December)			Average		
	NC	PY	XHK	NC	PY	XHK	NC	PY	XHK	NC	PY	XHK	NC	PY	XHK
WT	14.23 ± 0.05^a^	12.98 ± 0.40^b^	13.00 ± 0.17^b^	30.12 ± 0.65^a^	30.38 ± 0.41^a^	28.13 ± 0.38^b^	22.23 ± 0.23^ab^	23.83 ± 0.82^a^	21.73 ± 0.26^b^	6.33 ± 0.96^a^	15.32 ± 0.34^b^	10.20 ± 0.27^a^^b^	18.23 ± 0.21^a^	20.63 ± 0.20^b^	18.27 ± 0.07^a^
DO	11.12 ± 0.85^a^	8.83 ± 0.12^b^	8.86 ± 0.30^b^	9.64 ± 0.17^a^	7.83 ± 0.64^b^	8.66 ± 0.35^b^	11.88 ± 0.17^a^	8.19 ± 0.21^a^^b^	7.67 ± 0.26^b^	8.72 ± 0.50^a^	7.95 ± 0.95^b^	7.62 ± 0.23^b^	10.34 ± 0.25^a^	8.20 ± 0.28^b^	8.20 ± 0.07^b^
NO_3_-N	0.81 ± 0.03^a^	0.52 ± 0.07^b^	0.70 ± 0.11^ab^	0.51 ± 0.04^a^	0.22 ± 0.02^b^	0.18 ± 0.04^b^	0.63 ± 0.03^a^	0.30 ± 0.05^b^	0.37 ± 0.10^b^	0.53 ± 0.05^a^	0.35 ± 0.06^b^	0.39 ± 0.05^b^	0.62 ± 0.03^a^	0.35 ± 0.03^b^	0.41 ± 0.05^b^
NH_4_-N	0.21 ± 0.02^a^	0.05 ± 0.02^b^	0.03 ± 0.01^b^	0.14 ± 0.05^a^	0.07 ± 0.02^a^^b^	0.05 ± 0.01^b^	0.10 ± 0.02^a^	0.06 ± 0.01^a^^b^	0.02 ± 0.00^b^	0.04 ± 0.02^a^	0.06 ± 0.01^b^	0.04 ± 0.02^a^	0.12 ± 0.02^a^	0.06 ± 0.01^a^^b^	0.03 ± 0.01^b^
pH	7.95 ± 0.23^a^	7.13 ± 0.17^b^	7.19 ± 0.18^b^	8.68 ± 0.12^a^	7.46 ± 0.13^b^	7.37 ± 0.21^b^	8.79 ± 0.07^a^	8.25 ± 0.43^a^	7.65 ± 0.07^b^	7.70 ± 0.16^a^	7.08 ± 0.10^b^	7.14 ± 0.10^b^	8.29 ± 0.10^a^	7.48 ± 0.13^b^	7.34 ± 0.08^b^
Transparency	1.15 ± 0.10^a^	1.62 ± 0.04^b^	2.68 ± 0.79^b^	0.82 ± 0.04^a^	3.00 ± 0.09^b^	1.15 ± 0.08 ^c^	1.37 ± 0.05^a^	2.30 ± 0.18^b^	2.68 ± 0.37^b^	1.57 ± 0.05^a^	2.13 ± 0.19^b^	2.12 ± 0.08^b^	1.22 ± 0.03^a^	2.26 ± 0.04^b^	2.16 ± 0.16^b^

There is a significant difference between the groups denoted by different letters

Table 2 presents the contribution of environmental factors to the variation in gut microbiota obtained by VPA. All the variation partitioning fractions were significant in the permutation test (*p* < 0.05). Among the environmental factors, pH explained the most variation in gut microbiota (43.41%), followed by NH_4_-N (43.14%), DO (38.34%), transparency (34.41%), NO_3_-N (28.28%), and WT (19.40%).

**Table 2:** The relative contributions of environmental factors to variation in gut microbiota

Environmental factors	Variance explained	Sig
pH	0.4341	0.001***
NH_4_-N	0.4314	0.001***
DO	0.3834	0.001***
Transparency	0.3441	0.003**
NO_3_-N	0.2828	0.001***
WT	0.19398	0.022*

Significance levels with *, **, and *** represent p-value < 0.05, 0.01, and 0.001 between groups, respectively (permutation test)

**Fig. 5:**
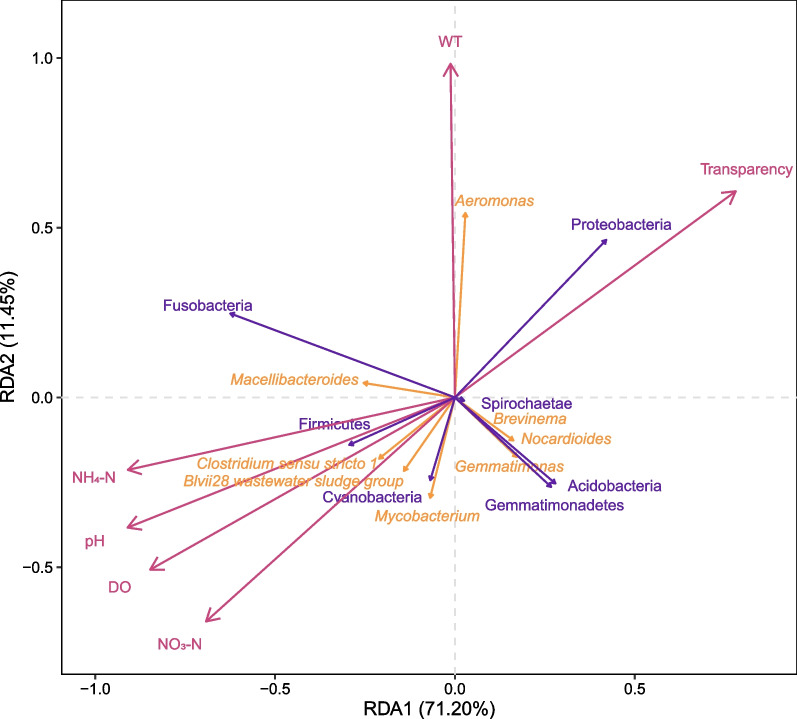
The correlation among samples, environmental factors, and significant microbes. Blue and yellow arrows indicate vectors of significant microbes at the phylum and genus levels, respectively, while red arrows represent vectors of environmental factors. A positive correlation is indicated when the angle between vectors is less than 90°, while a negative correlation is indicated when it is greater than 90°. The vectors are perpendicular to each other to indicate irrelevance

To further explore the correlation between environmental factors and significant microbes at the phylum and genus levels, redundancy analysis (RDA) was conducted, and the results are shown in Fig. 5. All six canonical axes explain 23.31% of the total variability. The first two axes, which contributed 71.20% and 11.45% of the explained variance (*p* < 0.01), respectively, were used for further analysis.

The results showed that Fusobacteria and *Macellibacteroides* were positively correlated with all environmental factors except transparency. Conversely, Spirochaetae, Acidobacteria, Gemmatimonadetes, *Brevinema*, *Gemmatimonas*, and *Nocardioides* exhibited an opposite trend. Firmicutes, Cyanobacteria, *Clostridium sensu stricto 1*, *Blvii28 wastewater sludge group*, and *Mycobacterium* positively correlated with NH_4_-N, pH, DO, and NO_3_-N, while exhibiting negative correlations with WT and transparency. On the contrary, Proteobacteria and *Aeromonas* showed the opposite trend.

## Discussion

This study demonstrates the impact of culture systems on the gut microbiota and metabolic profiles of bighead carp.

### Culture systems greatly influence the gut microbiota of bighead carp.

In the current study, the dominant phyla in bighead carp's gut microbiota were Proteobacteria, Fusobacteria, Actinobacteria, Bacteroidetes, and Firmicutes, consistent with previous findings in marine and freshwater fishes [11, 57–59] and supporting Luo's research on bighead carp [21]. Among all phyla, especially Cyanobacteria, it is considered to be directly consumed by filter-feeding fish [60]. As a filter-feeding planktivorous carp, bighead carp easily filter some colony-forming cyanobacteria [27, 61, 62]. The abundance of Cyanobacteria in the hindgut was significantly higher in NC than in the other two groups (Additional file 1: Fig S1C), possibly due to differences in food sources in the three culture systems.

Alpha and beta diversity are important parameters used to evaluate the structural characteristics of microbiota. In our study, gut microbiota diversity and richness were higher in alpine cold-water reservoirs and inner lakes than in ponds, consistent with other research [9, 11, 63, 64]. This could be due to larger habitats providing a wider range of diets. Therefore, fish may be constantly exposed to more bacteria [65].

Beta diversity analysis revealed significant differences in the gut microbial communities of bighead carp in all three culture systems, in agreement with other studies [9, 11]. This may be due to culture environments, diet, and genetics [66]. The VPA results showed that environmental factors explained up to 43.41% of the variation in the gut microbiota, which indicates a great influence of environmental factors. Diet is considered a critical factor in regulating the gut microbiota composition [67], and different bait provided by different culture systems could be an important reason for the differences in gut microbiota diversity. The bighead carp used in our study were from the same batch reared in the same hatchery and shared a similar genetic background, so the effect from the genetic background was relatively small. Therefore, we believe that differences in environmental factors and diet were the main causes of the significant differences observed.

LefSe analysis revealed 15 biomarkers that may cause significant differences in communities. At the phylum level, these included Fusobacteria, Firmicutes, Cyanobacteria, Proteobacteria, Spirochaetae, Acidobacteria, and Gemmatimonadetes; at the genus level, these included *Clostridium sensu stricto 1*, *Macellibacteroides*, *Blvii28 wastewater sludge group*, *Mycobacterium*, *Aeromonas*, *Brevinema*, *Gemmatimonas*, and *Nocardioides*. *Clostridium* is typically considered the most efficient lignocellulose degrader due to the presence of multienzyme complexes [68, 69] consisting of multiple cellulases and hemicellulases in combination with enzyme-free scaffoldin [70–72] that synergistically and efficiently degrade lignocellulose. They also contribute to the host's nutrition by supplying fatty acids and vitamins [73]*. Macellibacteroides* are capable of decomposing cellulose- and hemicellulose-derived sugars [74]. Several species of *Aeromonas* can produce cellulase [75] and have intensive cellulolytic activity [76, 77]. Although bighead carp are important plankton feeders [78], they lack the cellulase enzyme that breaks down the cell walls of algae in their gut [79]. We hypothesise that the cellulolytic action of these bacteria compensates for this deficiency in bighead carp and that bighead carp derive energy and nutrients from this process [27]. The abundance of both *Clostridium sensu stricto 1* and *Macellibacteroides* was positively correlated with the long and short diameter of muscle fibres (Spearman Rank Correlation). These may suggest that the culture systems improve nutrition levels and muscle quality in bighead carp by affecting gut microbes.

PICRUSt2 is a tool used for metagenome prediction to predict the approximate functional potential of a community [48]. In our study, 278 significantly different KEGG pathways were predicted, most of which were involved in metabolism, and organic systems, revealing the possible functional mechanisms of culture systems affecting gut microbes.

In conclusion, the culture systems significantly alter the community structure of gut microbiota and potentially impact their metabolic profiling.

### Culture systems have a significant impact on gut metabolic profiling.

Gut metabolites are the outcome of the joint metabolism of the host and microbial community and can reflect the outcomes of nutrient uptake, digestion, and absorption by the gut microbiota [80].

In this study, multivariate statistical analyses, including PCA and OPLS-DA, revealed significant changes in the intestinal metabolite profile of bighead carp under different culture environments, reflecting the significant influence of the environment on the joint metabolism of host and gut microbes.

Further exploration of the differential metabolites showed that 93 key metabolites were significantly enriched in 11 pathways, of which 4 were related to amino acid metabolism and 1 to translation, including "aminoacyl-tRNA biosynthesis", "arginine biosynthesis", "glycine, serine, and threonine metabolism", "alanine, aspartate, and glutamate metabolism", and "cysteine and methionine metabolism". The enriched pathways suggest that the culture systems exerted an important impact on host-microbe joint metabolism in the gut, particularly on amino acid metabolism and protein translation-related metabolic pathways.

Overall, the culture systems significantly altered intestinal metabolism.

### Potential association of fish muscle quality with microbes and metabolites in the gut

Several studies have demonstrated the beneficial role of gut microbes in improving muscle quality. For example, mice supplemented with *Lactobacillus plantarum* were accompanied by a change in muscle fibre type, that is, a significant increase in the proportion of type I fibres in the gastrocnemius muscle [13]. And when gut microbiota from obese Rongchang pigs and lean Yorkshire pigs were transferred to germ-free mice by faecal transplantation, mice fed Rongchang pig faeces tended to have increased body fat weight, increased percentage of slow muscle fibres, decreased diameter and cross-sectional area of the gastrocnemius muscle, and increased fat in gastrocnemius muscle compared to the mice fed Yorkshire pig faeces [14]. However, the mechanisms underlying these effects are not well understood.

Amino acids are important components of fish muscle quality [81]. Intestinal microorganisms regulate amino acids mainly through two mechanisms. On the one hand, microbes can utilise amino acids, producing acetic acid [82], propionic acid, butyric acid [83], hydrogen sulfide (H_2_S) [83, 84], polyamine [85], phenolic and indole compounds [86]. These metabolites play a crucial role in regulating host physiology [87]. However, this process exists in the large intestine and is largely not absorbed by the colonic mucosa [88], making it difficult to influence the host's amino acid metabolism. On the other hand, intestinal microbes can synthesise amino acids de novo [89]. Numerous reports of microbes synthesising amino acids affect the host's amino acid metabolism; for example, microbial lysine can be incorporated into host proteins, as observed in uremic patients and subjects consuming a low-protein diet [90, 91]. The significant contribution of microbial-derived lysine and threonine to free plasma lysine and threonine has also been observed in studies of nitrogen (protein)-sufficient diets in adults [89, 92]. In pigs fed diets incorporating 15N-NH_4_Cl and 14C-polyglucose, microbially produced amino acids such as valine, isoleucine leucine, phenylalanine, and lysine were found to be incorporated into human proteins [93] and absorbed mainly from the small intestine [93, 94]. In the present study, we found that L-cysteine, L-lysine, and L-threonine differed significantly among the three groups (Kruskal-Wallis test, FDR < 0.05) (Additional file 3: Fig S3A, S3B, S3C), and their levels were positively correlated with muscle quality. We suggest that these amino acids may be synthesised de novo by gut microbes and absorbed by the host, affecting the changes in amino acids in fish muscle and thus improving the quality of fish muscle.

Based on this hypothesis, we established the association between gut microbes and relevant metabolites using Spearman Rank Correlation with a cutoff of *r* > 0.65 and consequently inferred which microbes perform the corresponding functions using the R corrr package. According to the analysis, several bacteria, including *uncultured Chloroflexi bacterium*, *Pedobacter*, *Azohydromonas*, *Sinomonas*, *Patulibacter*, *Sorangium*, *Altererythrobacter*, and *Bryobacter,* may be potential amino acid-synthesising bacteria, as shown in Additional file 4: Fig S4. However, it's important to note that these findings are based on inference and correlation, and further experimental validation would be necessary to confirm these associations.

Additionally, certain metabolites may contribute to improving muscle quality in fish, such as glutamine which can increase the activity of the mammalian target of rapamycin (mTOR), a protein kinase that regulates protein synthesis in animal tissues and cells [95]. Although not yet available for fish [96], our study found that glutamine was the most abundant metabolite in the XHK group, followed by PY and NC (Additional file 3: Fig S3D), which is consistent with the muscle quality revealed by muscle microstructure. This suggests that glutamine could be a potential metabolite to enhance muscle quality in fish.

The flavour is one of the most important factors in influencing the edible quality of fish [97]. Nucleotides are a taste-active substance [98], among which the nucleotides inosine-5'-monophosphate (5'-IMP) and adenosine-5'-monophosphate (5'-AMP) contribute significantly to umami taste. They work in synergy with glutamate to intensify the taste sensation by binding to the same receptors, taste receptor type 1 members 1 and 3 [99, 100]. In our previous study, bighead carp in XHK exhibited a stronger umami intensity compared to other groups [12]. IMP is also considered an umami substance for fish products [101]. In the current study, 5'-IMP and glutamate followed the same trend of differences among the groups (Kruskal-Wallis test, FDR < 0.05) (Additional file 3: Fig S3E, S3F). This indicates that 5'-IMP and glutamate are probably the metabolites responsible for making the muscle of bighead carp tastier.

These findings suggest that gut microbiota and metabolites may play important roles in determining muscle quality in fish. This provides a practical direction for future research to improve the muscle quality of bighead carp. To verify the role of these gut microbes and metabolites, we may isolate and extract intestinal flora and conduct transplantation in aseptic mice, as well as supplement metabolites.

## Conclusions

It is evident that culture systems significantly impact the intestinal microbiota and metabolites of bighead carp. Our findings demonstrate that the culture systems not only alter the gut microbial community of bighead carp, with notable variations in community structure, abundance, and potential metabolic functions, but also affect host gut metabolism, particularly with significant enrichment in pathways related to amino acid metabolism. Moreover, we discuss potential mechanisms that may impact muscle quality. We hypothesize that significantly different amino acids in the gut are the primary cause of the effect on muscle quality. Based on this hypothesis, we suggest that gut microbes may play a role in altering muscle quality in fish through the biosynthesis of amino acids.

Further investigation into the potential association between fish muscle quality and gut microbes and metabolites can provide a foundation for enhancing bighead carp's muscle quality and nutritional value.

## Supplementary Information

**Additional file 1**. **Fig S1**. The relative abundance of indicator taxa at the phylum level. (A) Fusobacteria. (B) Firmicutes. (C) Cyanobacteria. (D) Proteobacteria. (E) Spirochaetae. (F) Acidobacteria. (G) Gemmatimonadetes. Significance levels with *, **, and *** represent FDR < 0.05, 0.01, and 0.001 between groups, respectively (Kruskal-Wallis test).

**Additional file 2**. **Fig S2**. The relative abundance of indicator taxa at the genus level. (A) *Clostridium sensu stricto 1*. (B) *Macellibacteroides*. (C) *Blvii28 wastewater sludge group*. (D) *Mycobacterium*. (E) *Aeromonas*. (F) *Brevinema*. (G) *Gemmatimonas*. (H) *Nocardioides*. Significance levels with *, **, and *** represent FDR < 0.05, 0.01, and 0.001 between groups, respectively (Kruskal-Wallis test).

**Additional file 3**. **Fig S3**. Normalised peak intensity of potential metabolites which influence fish muscle quality (A) L-cysteine. (B) L-lysine. (C) L-threonine. (D) glutamine. (E) 5’-IMP. (F) L-glutamate. Significance levels with *, **, and *** represent FDR < 0.05, 0.01, and 0.001 between groups, respectively (Kruskal-Wallis test).

**Additional file 4**. **Fig S4**. Potential microbes synthesising amino acids. The curves represent the correlation between metabolites and microbes, with greener colours representing stronger positive correlations and yellower colours representing stronger negative correlations.

**Additional file 5**. **Table S1.** The abundance and taxonomy of OTUs.

**Additional file 6**. **Table S2.** The abundance and significance of top 10 microbes at the phylum level.

**Additional file 7**. **Table S3.** The abundance and significance of top 10 microbes at the genus level.

**Additional file 8**. **Table S4.** The abundance and classification of metabolites.

**Additional file 9**. **Table S5.** The parameters and evaluation of PCA and OPLS-DA models.

**Additional file 10**. Table S6. The significance and impact of enriched pathways.

**Additional file 11**. Table S7. The potential metabolites and microbes at the phylum level.

**Additional file 12**. Table S8. The potential metabolites and microbes at the genus level.

## Data Availability

The 16S sequencing data were deposited into the National Center of Biotechnology Information (NCBI) Sequence Read Archive (SRA) database under Accession PRJNA933520. Data generated or analysed during this study are included in this published article and its supplementary information files.

## Abbreviations

NC: Nancheng
PY: Poyang
XHK: Xiahuikeng
DNA: Deoxyribonucleic acid
WT: Water temperature
DO: Dissolved oxygen
NO_3_-N: Nitrate nitrogen
NH_4_-N: Ammonium nitrogen
16S rRNA: 16S ribosomal ribonucleic acid rRNA
PCR: Polymerase chain reaction
QIIME: Quantitative insights into microbial ecology
OTU: Operational taxonomic units
NMDS: Non-metric multidimensional scaling
ANOSIM: A one-way analysis of similarity
LEfSe: Linear discriminant analysis Effect Size
LDA: Linear discriminant analysis
FDR: False discovery rate
PICRUSt2: Phylogenetic investigation of communities by reconstruction of unobserved states
LC–MS: Liquid chromatograph–mass spectrometer
GC–MS: Gas chromatograph–mass spectrometer
QC: Quality control
MS: Mass spectrum
RSD: Relative standard deviation
PCA: Principal component analysis
OPLS-DA: Orthogonal projections to latent structures-discriminant analysis
VIP: Variable importance in projection
MetPA: Metabolic pathway analysis
VPA: Variation partitioning analysis
RDA: Redundancy analysis
