# Supplementary material for: The impact of culture systems on the gut microbiota and gut metabolome of bighead carp (Hypophthalmichthys nobilis)
Source: Anim Microbiome. 2023 Apr 1;5:20. doi: 10.1186/s42523-023-00239-7 (PMC10067185; doi:10.1186/s42523-023-00239-7)
Supplement: Supplementary file 1 — Additional file 1. Fig S1. The relative abundance of indicator taxa at the phylum level. (A) Fusobacteria. (B) Firmicutes. (C) Cyanobacteria. (D) Proteobacteria. (E) Spirochaetae. (F) Acidobacteria. (G) Gemmatimonadetes. Significance levels with *, **, and *** represent FDR < 0.05, 0.01, and 0.001 between groups, respectively (Kruskal-Wallis test). [file 42523_2023_239_MOESM1_ESM.docx]

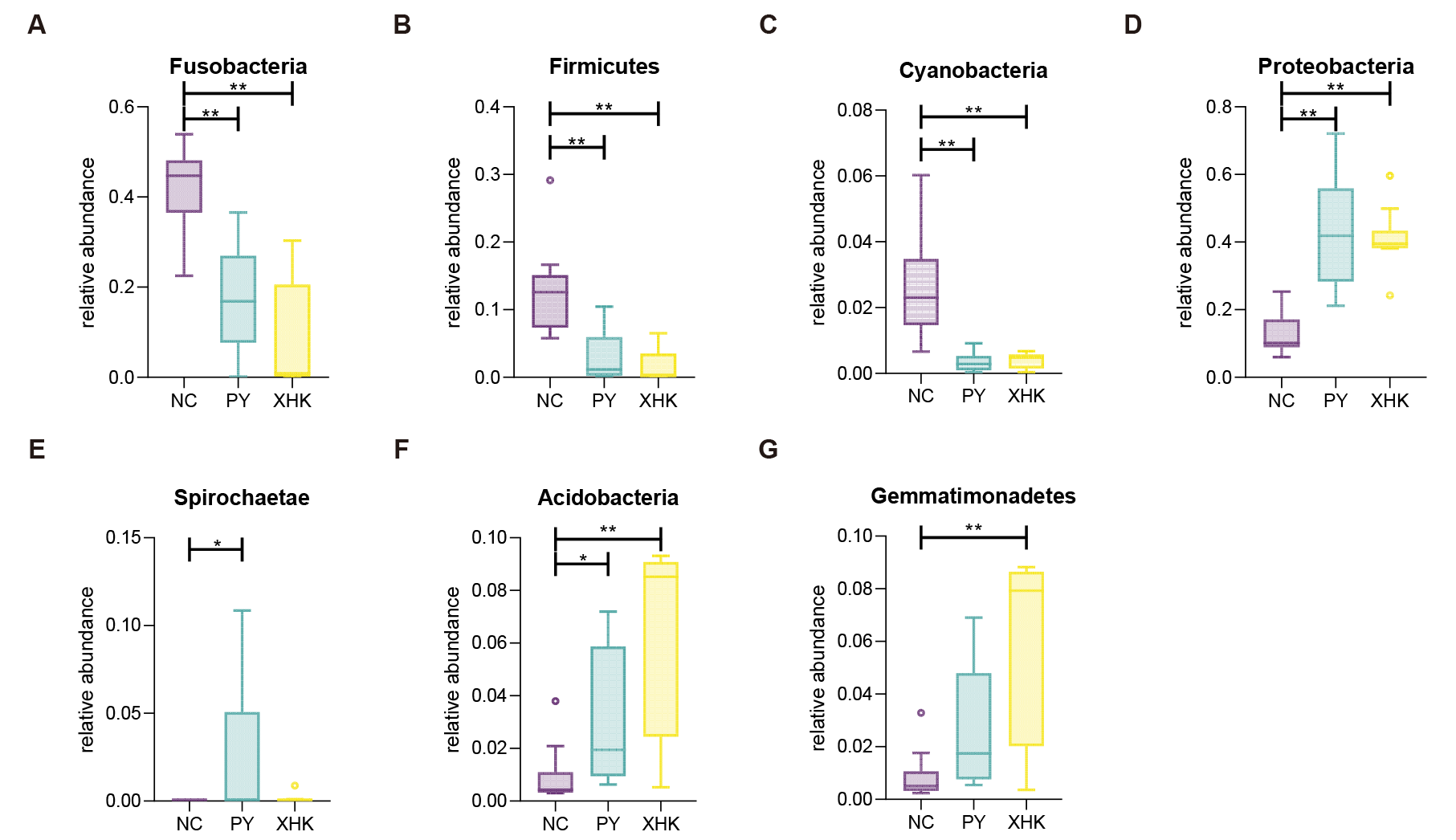


**Fig S1.** The relative abundance of indicator taxa at the phylum level. (A) Fusobacteria. (B) Firmicutes. (C) Cyanobacteria. (D) Proteobacteria. (E) Spirochaetae. (F) Acidobacteria. (G) Gemmatimonadetes. Significance levels with *, **, and *** represent FDR < 0.05, 0.01, and 0.001 between groups, respectively (Kruskal-Wallis test).
