# Supplementary material for: The impact of culture systems on the gut microbiota and gut metabolome of bighead carp (Hypophthalmichthys nobilis)
Source: Anim Microbiome. 2023 Apr 1;5:20. doi: 10.1186/s42523-023-00239-7 (PMC10067185; doi:10.1186/s42523-023-00239-7)
Supplement: Supplementary file 2 — Additional file 2. Fig S2. The relative abundance of indicator taxa at the genus level. (A) Clostridium sensu stricto 1. (B) Macellibacteroides. (C) Blvii28 wastewater sludge group. (D) Mycobacterium. (E) Aeromonas. (F) Brevinema. (G) Gemmatimonas. (H) Nocardioides. Significance levels with *, **, and *** represent FDR < 0.05, 0.01, and 0.001 between groups, respectively (Kruskal-Wallis test). [file 42523_2023_239_MOESM2_ESM.docx]

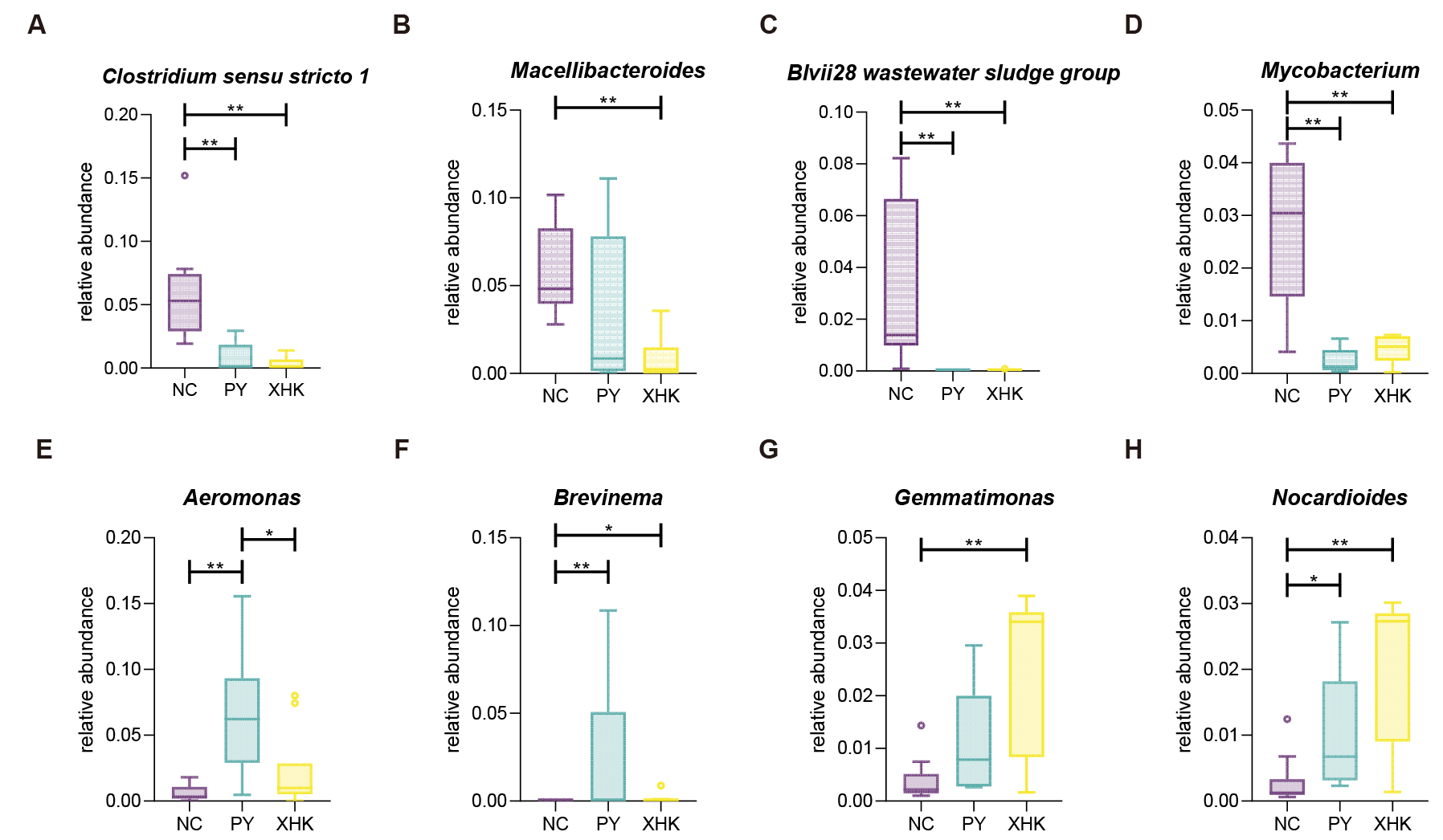


**Fig S2.** The relative abundance of indicator taxa at the genus level. (A) *Clostridium sensu stricto 1*. (B) *Macellibacteroides*. (C) *Blvii28 wastewater sludge group*. (D) *Mycobacterium*. (E) *Aeromonas*. (F) *Brevinema*. (G) *Gemmatimonas*. (H) *Nocardioides*. Significance levels with *, **, and *** represent FDR < 0.05, 0.01, and 0.001 between groups, respectively (Kruskal-Wallis test).
