# Supplementary material for: The impact of culture systems on the gut microbiota and gut metabolome of bighead carp (Hypophthalmichthys nobilis)
Source: Anim Microbiome. 2023 Apr 1;5:20. doi: 10.1186/s42523-023-00239-7 (PMC10067185; doi:10.1186/s42523-023-00239-7)
Supplement: Supplementary file 3 — Additional file 3. Fig S3. Normalised peak intensity of potential metabolites which influence fish muscle quality (A) L-cysteine. (B) L-lysine. (C) L-threonine. (D) glutamine. (E) 5’-IMP. (F) L-glutamate. Significance levels with *, **, and *** represent FDR < 0.05, 0.01, and 0.001 between groups, respectively (Kruskal-Wallis test). [file 42523_2023_239_MOESM3_ESM.docx]

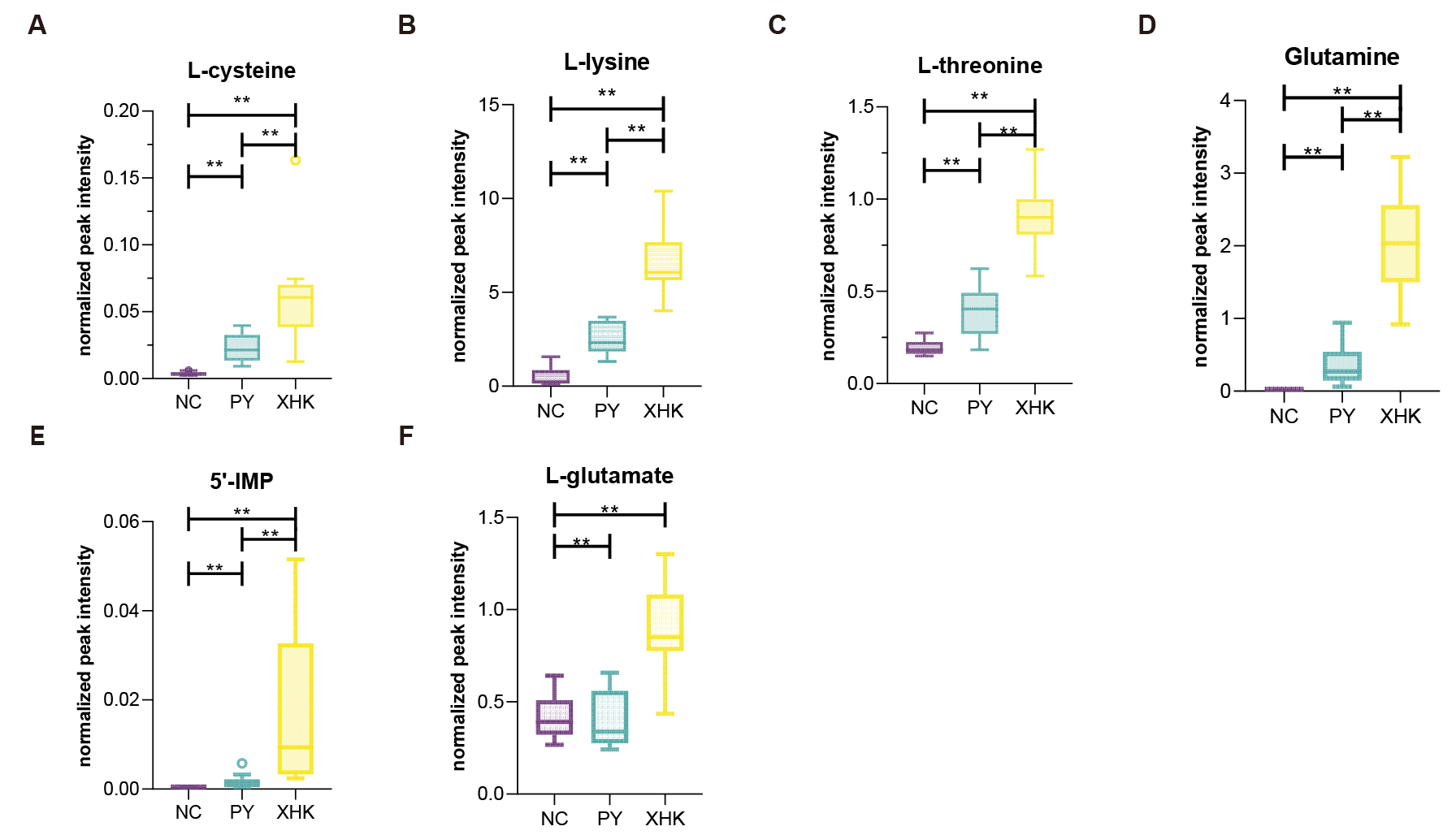


**Fig S3.** Normalised peak intensity of potential metabolites which influence fish muscle quality (A) L-cysteine. (B) L-lysine. (C) L-threonine. (D) glutamine. (E) 5’-IMP. (F) L-glutamate. Significance levels with *, **, and *** represent FDR < 0.05, 0.01, and 0.001 between groups, respectively (Kruskal-Wallis test).
