# Supplementary figures and images for: The impact of culture systems on the gut microbiota and gut metabolome of bighead carp (Hypophthalmichthys nobilis)
Source: Anim Microbiome. 2023 Apr 1;5:20. doi: 10.1186/s42523-023-00239-7 (PMC10067185; doi:10.1186/s42523-023-00239-7)

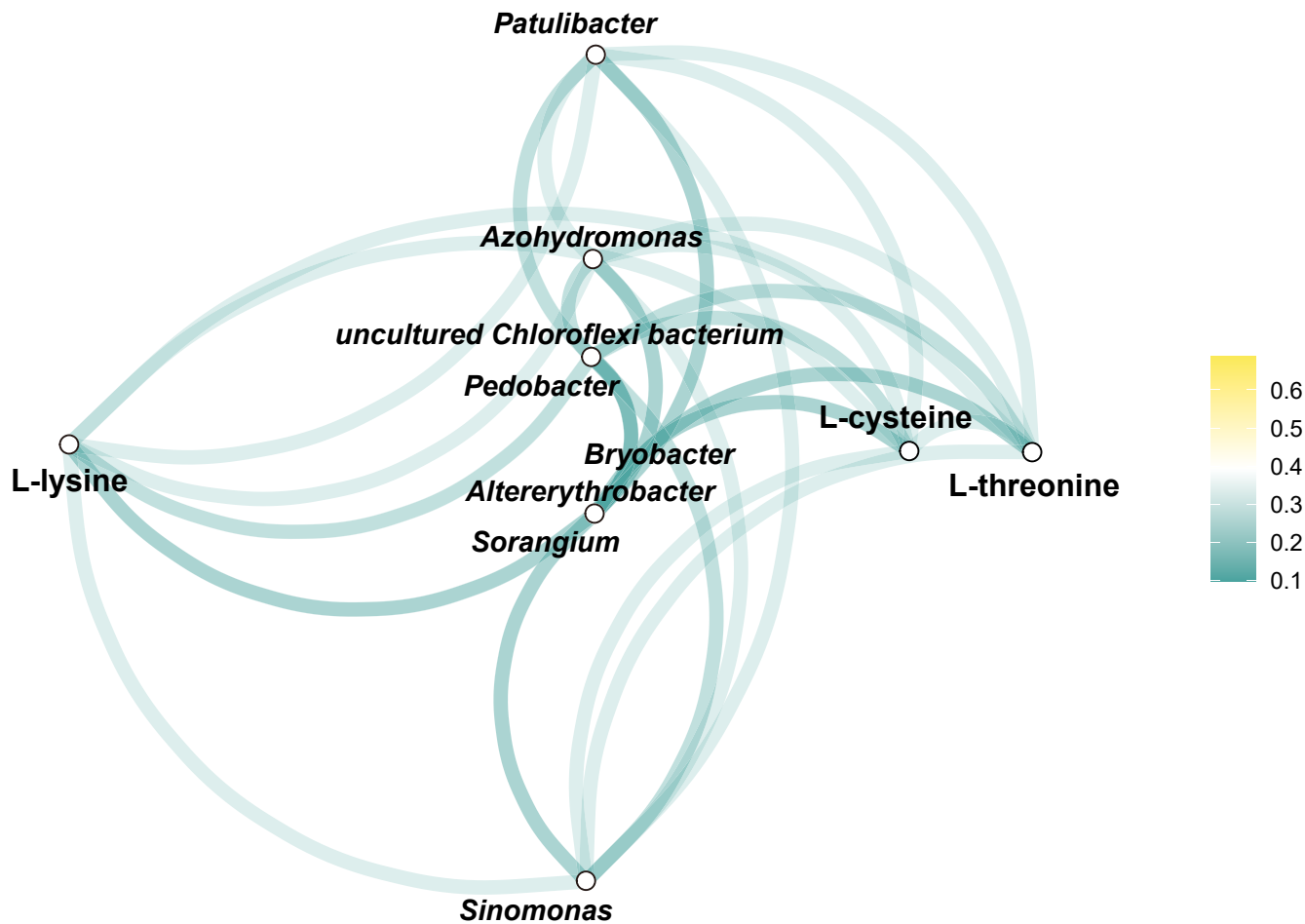

Supplement: Supplementary file 4 — Additional file 4. Fig S4. Potential microbes synthesising amino acids. The curves represent the correlation between metabolites and microbes, with greener colours representing stronger positive correlations and yellower colours representing stronger negative correlations. [file 42523_2023_239_MOESM4_ESM.pdf]
